# A Missing Switch in Peptide Exchange for MHC Class II Molecules

**DOI:** 10.3389/fimmu.2019.02513

**Published:** 2019-10-23

**Authors:** Christian Freund, Thomas Höfer

**Affiliations:** ^1^Laboratory of Protein Biochemistry, Institute for Chemistry and Biochemistry, Freie Universität Berlin, Berlin, Germany; ^2^Division of Theoretical Systems Biology, Deutsches Krebsforschungszentrum, Heidelberg, Germany

**Keywords:** MHC class II, HLA-DM, exchange catalyst, HLA-DO, switch

## Introduction

Antigen processing and loading of peptides onto MHC class II molecules is a multistep process that involves vesicular transport of the MHCII molecules along the secretory pathway, where they eventually merge with antigen-containing endocytic vesicles or phagosomes (1). It is within these late endosomal or lysosomal compartments that protein antigens become degraded by proteases, most prominently by cathepsins, and where catalyzed peptide exchange by HLA-DM fulfills its role in the efficient replacement of the invariant chain-derived peptide CLIP by high-affinity pathogen- or host cell-derived peptides. Protease action may be limited by protein antigen abundance and redox conditions, while HLA-DM is regulated at several stages, including by expression levels, pH, or the co-expression of the competitive inhibitor HLA-DO. HLA-DM activity leads to significant changes in the immunopeptidome of antigen-presenting cells, thereby tailoring T cell responses and often shifting antigenicity toward high-affinity immunodominant epitopes (2). Control of DM activity by DO has been described to be of prime importance in thymic epithelial cells, in a subset of dendritic cells, and in B cells when entering the germinal centers for affinity maturation and class switching (3, 4). In all of these cases, the switch from a broader, self-peptide (CLIP) dominated immunopeptidome to a more focused repertoire is necessitated by the requirement for more stringent antigen presentation, often preceding more intense T cell reactivity and proliferation. Here, we review data on this cellular switch in the functionality of antigen presentation and propose that it is promoted by an as yet poorly understood molecular switch. Acknowledging that general biophysical parameters such as pH and redox are important for antigen processing in general, an elusive DM-DO switch is postulated that would allow rapid and strong shifts in immunopeptidomes. We capitalize on theoretical considerations to back our opinion that a regulatable switch would have the advantage of allowing for a rapid and possibly signal-dependent change in the peptide selection process, as might be required in the context of rapidly changing immunological conditions.

## Regulation of Antigen Processing

Proteolysis of antigens for MHC presentation and T cell surveillance, while certainly modulated in its efficiency, is thought to constantly report on the proteome state of cells and organs in the body. It is mostly viruses, bacteria, and cancer cells that have developed strategies to counter the expression of MHCI or the presentation of antigens by MHC class I molecules, thereby tuning down the corresponding MHCI immunopeptidomes. For MHC class II, the situation is more complex. Presentation, in this case, is restricted to certain types of immune cells, and MHC class II expression itself is regulated depending, e.g., on the maturation state of a certain cell type (1). Furthermore, molecules associated with effective MHCII peptide presentation, such as cathepsins, the exchange catalyst HLA-DM, or its inhibitor, HLA-DO, have been shown to be regulated in their expression (4). Furthermore, the function of these proteins is pH-dependent, with an optimum of activity (e.g., for certain cathepsins or the exchange factor HLA-DM, the pH optimum is close to the acidic pH of the late endosome). We note that HLA-DM activity differs largely for MHCII allotypes and thus that DM susceptibility can be truly defined only with regard to a specific peptide-MHCII complex (5). For example, in the mouse system it has been shown that the I-Ab or I-Ad alleles are strongly dependent on the mouse homolog of HLA-DM, H-2M, while the E-Ad and E-Ak variants are not (6, 7). However, it seems that even for the latter variants, the exact composition of the antigen repertoire can be modulated by H-2M (7). Furthermore, for humans it has been found that several variants of DM exist. Certain combinations of the α- and β-chain of DM are expressed in the population, and the biochemical and cellular properties of these DM proteins differ measurably with regard to their activity and pH dependence and thus result in distinct immunopeptidomes (8, 9).

It has been proposed that redox conditions within the phagolysosomal and endolysosomal compartments are critical for antigen presentation (10). Here, a balance has to be maintained between rendering the cysteines of proteases in a reduced, active form and not reducing the essential disulfide bonds of other proteins as they are present, for example, in the MHCII molecules themselves. This leaves the conundrum of how certain antigens can be processed that are stabilized by disulfide bonds but need to be reduced for efficient digestion by proteases. It appears that enzymes, such as the gamma-interferon-inducible lysosomal thioreductase (GILT), play an important role (11). For example, it has been shown that reduction of the house dust mite allergen Derp1 depends on GILT and that GILT thereby leads to more efficient processing of the protein. Consequently, in a mouse airway inflammation model of asthma, GILT knockout mice exert mitigated allergic responses (12). Independent of disulfide reduction, the foldedness of the birch pollen allergen Betv1 along the endolysosomal pathway has been shown to be critical for its immunogenicity (13), indicating that protein stability is certainly one of the parameters that determine the degradation kinetics of a protein and its subsequent loading onto MHCII molecules. The generally high thermodynamic stability of long-lived MHCII-peptide complexes might be one reason why they are largely protected from degradation themselves. However, why the more instable MHCII allotypes such as certain HLA-DQ variants are shielded from degradation is not clear; presumably membrane partitioning and nanoscale localization within the late endosome contribute to protecting them.

While the importance of pH, redox conditions, and protein stability for antigen presentation is undisputed, these parameters describe general biophysical properties that are not subject to acute control. They rather shape the constitutive process of presenting peptidomes on MHCII and may tune its general features. Modulation of this constitutive MHCII pathway by changes in the gene expression of its critical components could impose long-term control that might be required, for example, during the differentiation of dendritic cells or B cells. However, the rapid switches of MHCII presentation induced by antigen- or pathogen-related signals, such as those delivered by Toll-like- or B-cell receptors (3), are unlikely to rely solely on comparatively slow changes in gene expression (typically requiring many hours to become manifest at protein level). Hence, we hypothesize that a molecular switch, operating on shorter “biochemical” time scales of minutes to hours, is involved in the timely changes of the presented peptides. First, a cell might signal strong receptor engagement in order to promote the general turnover of antigen, thereby adjusting its presentation properties to the new conditions. Thus, sustained exposure to antigen would be distinguished from more serendipitous events and result in a robust response that precedes irreversible fate decisions. Molecular switches of this kind would typically operate at the level of transport or proteolysis. Secondly, a molecular switch could be engaged at the repertoire level, changing the composition of the immunopeptidome and thereby directly altering putative T cell responses. This type of switch could comprise site-specific proteolytic events or modulators of the peptide exchange process itself. In both cases, altered activity might lead to a shift in the ratio of self-peptides to pathogen-derived antigen and thus provide a means of rapidly modulating the activation of T cells. In particular, regulation of the peptide exchange process itself as the most downstream event in the processing pathway seems to be well-suited to ultimately tuning the presentation of antigen and stimulation of T cells.

## Control of Antigen Exchange by HLA-DM

**Figure 1:**
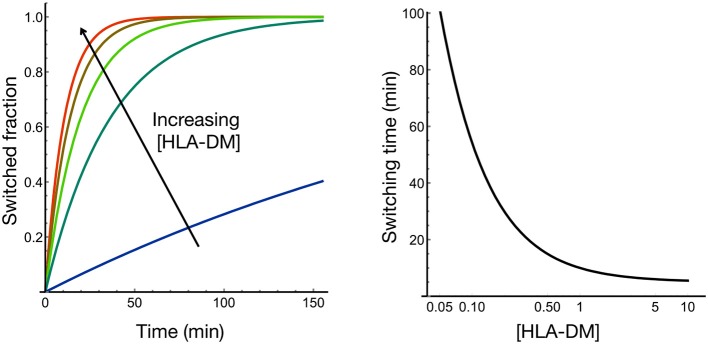
HLA-DM concentration controls the rate of peptide exchange at HLA. We modeled how rapid transitions in the conformational ensemble of HLA-peptide are modulated by HLA-DM binding; HLA-DM stabilizes a rare active conformational state that facilitates peptide exchange [see Figure 1E in Wieczorek et al. (22)]. Based on the experimental data obtained from Wieczorek et al. (22), the modeling shows that HLA-DM concentration controls the switching time most strongly in the substoichiometric regime.

Antigen loading of MHC class II molecules is a process that depends on the general features of endolysosomal processing but also capitalizes on molecules uniquely evolved to enable the highly efficient exchange of placeholder peptides against exogenous, often pathogen-derived antigens in professional antigen-presenting cells. The placeholders, termed class II-associated invariant chain peptides, are derived from the invariant chain (Ii or CD74) (14) as peptides of different length displaying distinct properties (15, 16) and, for most HLA allotypes, bind via the core sequence MRMATPLLM to MHCII molecules. CLIP binding to human and mouse MHCII molecules differs widely for individual allotypes (17), and several mouse alleles (I-Ak, I-Ed, I-Ek) (6, 7), as well as human allotypes (e.g., HLA-DQα1^*^0501/DQβ1^*^0301) (18, 19), show poor CLIP binding, and thus peptide replacement takes place efficiently even in the absence of H-2M/HLA-DM. However, the exchange catalyst for these alleles still seems to be required to stabilize unoccupied MHCII molecules or efficiently shape the repertoire toward higher affinity peptides (7). Interestingly, the CLIP sequence can bind in two flipped orientations along the MHCII binding groove, with the equilibrium of the canonical vs. inverted binding mode depending on the length of the N- and C-terminal overhang regions, a process that itself is catalyzed by HLA-DM (20, 21). In any event, the CLIP peptides of HLA-DM/H-2M susceptible allotypes are replaced in the late endosomal compartments by other peptides with similar or higher affinity. Alternatively, if large concentrations of a non-optimal antigen are provided, it might also be loaded onto the MHCII molecule, simply based on the law of mass action. However, since many MHCII-CLIP complexes, especially those of the HLA-DR locus, are already of high kinetic stability, replacement rates are very low for these HLA allotypes and would not proceed to a significant degree on a physiologically relevant timescale (minutes-to-hours). The exchange catalyst HLA-DM, which has its activity optimum at or near the acidic pH of the late endosome, leads to more efficient exchange even for MHCII allotypes that have low CLIP off-rates (2). Peptide exchange occurs via rare conformation in the HLA molecules, which occupy at most a few percent of the conformational ensemble and are recognized by HLA-DM (22, 23). This mechanism ensures that, for stable pMHCII complexes, the peptide can still be replaced by higher affinity ligands within a time frame of seconds to minutes. A simple mathematical model based on experimentally determined exchange rates (22) shows how the HLA-DM to HLA ratio controls the switching time (Figure 1). A somewhat sub-stoichiometric ratio of DM:MHCII in the range of 0.1–0.3 still enables switching times within the order of minutes (Figure 1). Thus, the active concentration of the peptide exchange catalyst HLA-DM controls switching time.

Indeed, the ratios of DM to MHCII in primary cells have been reported to be quite variable and to depend on the differentiation state of professional antigen-presenting cells (16). However, the curve in Figure 1 also argues that the system does not need to operate at a stoichiometric steady-state level for efficient catalysis, leaving open the question to what extent expression levels have to change in order to modulate the presented repertoire significantly. Experimentally, it has been shown that a significant minority of peptides and epitopes change when DM levels are raised from low to high in a cellular model (2). In particular, the amount of high affine peptides is fostered in the presence of HLA-DM, a consequence that might be favorable in a certain immunological context but undesired in others. Therefore, regulation of DM activity is a key issue when considering global shifts in immunopeptidomes during MHCII-mediated T cell responses.

## Discussion–Switching off Catalyzed Peptide Exchange

Biasing the repertoire of MHCII bound peptides toward high-affinity ligands might be harmful or advantageous, depending on the immunological context [reviewed in Alvaro-Benito et al. (24)]. For example, it has been found that the absence of HLA-DM in mice in the context of a type I diabetes model prevents the animals from acquiring the disease (25), while, on the other hand, DM seems to be required for constraining bacterial pathogens such as *Mycobacterium tuberculosis* (26). Moreover, DM expression in the thymus has been found to be low in the cortical but high in the medullary epithelial cells of the thymus (27), indicating that positive and negative selection have distinct requirements for DM during T cell development. Apart from the modulation of DM activity during these processes by regulation, DM gene expression downregulation of HLA-DM can also be achieved by co-expression of the DM competitive inhibitor HLA-DO (DO). DO binds with much higher affinity to DM than canonical MHCII molecules, thereby fully abrogating DM exchange activity when present at stoichiometric concentrations (28, 29). DO has also been suggested to have a direct effect on classical MHC class II molecules by recognizing a receptive conformation of the common allotype HLA-DR1 (30), and it will be interesting to see whether the underlying rare conformations can be detected directly by experiment. More recently, a comprehensive immunopeptidome study was performed comparing DO knockout and wild-type human lymphoblastoid HLA-DR1 homozygous LG-2 cell lines, extending previous studies on DM-independent peptide loading (6, 7) and corroborating the finding that DO broadens the repertoire, thereby counteracting the effect of DM to a certain extent (4). How far such broadening of the repertoire plays a role during certain phases of murine B cell development and dendritic cell differentiation, two processes where DO expression is known to be high, is an intriguing question. In human B cells, DO levels are high only when B cell development in the bone marrow is complete and are then downregulated in germinal centers, where affinity maturation of B cells proceeds and where T/B cell cooperation becomes of critical importance (31, 32). March9-mediated ubiquitination is mostly thought to be responsible for the reduced DO levels in GC B cells (33), but this leaves open the question of how March9 activity is itself regulated. The DM-DO complex is extremely stable and pH insensitive when investigated *in vitro* (K_D_ = 3.7 nM). However, once dissociated from DM, DO is rapidly inactivated at acidic pH, and an indirect influence of acidification on DO stability along the endolysosomal pathway has therefore been suggested (34).

The degradation rate of DO can be modeled based on the K_D_ of the complex. Assuming a typical k_on_ for protein-protein interactions of 10^5^ M^−1^ s^−1^ (35), the calculated half-time for complex dissociation is ~30 min. Assuming that each dissociation event will translate into conformational changes and subsequent degradation of the protein, this time represents the lower limit for DO downregulation. In cells, there will be some degree of competition between fast rebinding of acutely dissociated DO to DM and DO degradation, so that the effective half-life for DO is likely to be longer. Indeed, *in vitro* studies showed that concentrations of free DM and its associated exchange activity after preincubation of DM:DO complexes at acidic pH were significant only after 2 h and free DM was still increasing after 24 h when analyzing loading of the hemagglutinin peptide HA in cellular lysates (34). Thus, it seems that acidification alone would lead to a slow, gradual, and non-reversible increase in DM activity.

Rather than degradation of DO after slow release from a tight complex with DM, the lowering of the effective DO-DM affinity would provide a much faster means to release DM activity from DO inhibition and, in turn, switch the HLA-presented peptidome. Moreover, this switch would be reversible as long as DO is present, enabling the system to adapt to changing environmental stimuli. What is the evidence that such a switch would be advantageous *in vivo*? There is no direct evidence yet that the DM-DO complex is reversibly and rapidly switchable. However, circumstantial findings for adaptive changes in antigen presentation exist where fast control of the DM:DO ratio would be the most straightforward manipulatable parameter to change the peptide repertoire. As mentioned before, DO expression is seen in B cells first when they transition from the immature to the mature state. These cells then enter the germinal centers, and as earlier studies by Jensen and coworkers (32) showed, stably express DO in the initial IgD^+^CD38^−^ B cell population that has not yet undergone affinity maturation. Interestingly, GC B cells of the IgD^−^CD38^+^ type, which should consist of centrocytes and centroblasts, downregulate DO (32). Since these cells, especially the centrocytes in the light zone, are in intense contact with follicular TFH cells, DO downregulation is anticipated to unleash DM activity in order to allow the display of high-affinity pMHCII complexes that are in turn prone to engage more robustly in sustained T cell activation and thus provide B cell help. Interestingly, a third GC B cell population, namely IgD^−^CD38^−^, thought to represent memory cells, shows robust DO expression levels. Thus, it is clear that interconverting B cell populations exist that are under selective pressure to encounter high-affinity antigen (36). While changing DO levels reflect this requirement, it is likely that the genetic control is supported by regulation at the protein level. Ubiquitination-dependent degradation surely represents a possible regulatory mechanism (33), but it has the disadvantage of being irreversible and energetically costly. A reversible switch would, for example, allow the individual GC B-cell populations to interconvert prior to an irreversible fate decision. Moreover, when coupled to B cell receptor activation, a more robust “two-signal” scenario could be envisaged, where B cell receptor signaling by a higher affine B cell receptor is intertwined with more persistent presentation of a high-affinity pMHCII complex. There is experimental evidence that DM interacts with endocytosed B cell receptor in the late endosome (37), and it is thus not unlikely that such a coupling exists. It will be important to test whether the DM/Ig binding is altered in the presence of DO, since this could indicate that the BCR could act as a switch itself or at least contribute to a more acute response during B cell antigen presentation.

What could be the nature of this switch? Is it possible that a proteolytic event is coupled to such a switch in activity? It is well-known, for example, that invariant chain processing is a processive event where cleavage proceeds from the C-terminal to the N-terminal end (38–40). In particular, Cathepsin-S is critical for the production of the N-terminal fragments (41, 42) that could in principle be involved in the appearance of peptides other than CLIP and that may exert unanticipated functions through binding to canonical or non-canonical MHCII molecules. The fact that at least a partial MHCII binding groove exists in DO makes it at least conceivable that a proteolytically cleaved peptide binds to it (28).

This peptide would have been identified if it was constitutively present or if several peptides could bind via anchor residues as is the case for canonical MHCII molecules. Rather, this peptide would be produced under certain conditions in a switch-like manner that also allows its fast removal once DM is bound again or when ubiquitination-mediated degradation ensues. The proteolytic hypothesis, stating that inducible cleavage of a protein fragment results in a competitive DO binder, could be tested by applying selective inhibitors against, for example, cathepsin S (43) and analyzing the amount of free vs. bound HLA-DM. Alternatively, the spectrum of post-translational modifications of free vs. DO-bound DM molecules could be revisited, as there is ubiquitination, phosphorylation, glycosylation, and lipid modification. Notably, the DM α-chain contains a putative palmitoylation site in its short cytoplasmic tail (44) that could serve as a signal in changing the nanoscale localization of the molecule. Similarly, glycosylation and phosphorylation sites have been identified in the DO β-chain (45, 46). Capitalizing on modern, highly sensitive mass spectrometers, previously unobserved changes might be captured that are physiologically relevant and that could be validated by corresponding site-specific mutations. Together with the option that Ig binding could also act in a manner dependent on DM-DO complex-formation (37) (see above), we thus present three experimentally testable conditions to reinforce or disprove the suggestions made in this opinion article.

Assuming the existence of such a molecular event, it will quickly release inhibition of DM by DO and thus will lead to a temporally regulatable activity of the exchange catalyst. As the concentration of active DM not only controls the speed of peptide exchange but also affects the affinity range of presented peptides in an as yet poorly understood manner (see above), such a molecular switch would be an important regulator of the quality and quantity of antigen presentation by MHCII. It is worth pointing out that antigen recognition, at the T cell end of the interaction with the APC, also employs an active molecular switch: T cells do not simply sense the affinity of the interaction of peptide-loaded MHC with the T cell receptor but rather employ kinetic proofreading to discriminate between their agonists and self-peptides (47, 48). Thus, in a conceptual framework, the regulation of antigen presentation on the APC may not be dissimilar from antigen recognition by T cells in employing active molecular switches.

## Author Contributions

CF and TH drafted and wrote the manuscript together.

### Conflict of Interest

The authors declare that the research was conducted in the absence of any commercial or financial relationships that could be construed as a potential conflict of interest.
